# Redox regulation meets metabolism: targeting PRDX2 to prevent hepatocellular carcinoma

**DOI:** 10.1002/1878-0261.70194

**Published:** 2025-12-21

**Authors:** Naroa Goikoetxea‐Usandizaga, María Luz Martinez‐Chantar, Carolina Conter

**Affiliations:** ^1^ Center for Cooperative Research in Biosciences (CIC bioGUNE) Basque Research and Technology Alliance (BRTA) Derio Spain; ^2^ Centro de Investigación Biomédica en Red de Enfermedades Hepáticas y Digestivas (CIBERehd) Madrid Spain

**Keywords:** hepatocellular carcinoma, MASH, metabolism, Peroxiredoxin 2, ROS

## Abstract

Metabolic dysfunction‐associated steatohepatitis (MASH) is emerging as a major driver of hepatocellular carcinoma (HCC). Crouchet et al. identify PRDX2 as a key regulator linking oxidative stress, metabolic imbalance, and oncogenic signaling. Across multiple *in vivo* and *in vitro* models, PRDX2 inhibition restores metabolic homeostasis, reduces tumor initiation, and selectively impairs HCC cell survival. These findings highlight PRDX2 as a promising biomarker and hepatocyte‐directed target for chemoprevention, emphasizing the importance of the interplay between metabolism and liver cancer development.

## Graphical abstract

PRDX2 acts as a central redox hub linking metabolic dysfunction‐associated steatohepatitis (MASH) to hepatocellular carcinoma (HCC). In normal hepatocytes, PRDX2 maintains redox balance and metabolic homeostasis under oxidative stress. In contrast, during malignant transformation, PRDX2 promotes oncogenic signaling, stemness, and tumor initiation. Hepatocyte‐specific PRDX2 inhibition blocks HCC progression while preserving normal liver function, identifying PRDX2 as a potential therapeutic target.

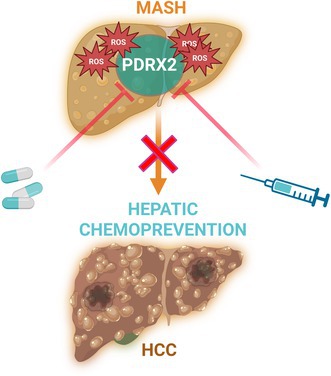

**Abbreviations**
AMPK: AMP‐activated protein kinase
CD44: cluster of differentiation 44
CTNNB1: catenin beta‐1 (β‐catenin)
DEN/CDA‐HFD: diethylnitrosamine/choline‐deficient, L‐amino acid–defined, high‐fat diet
FGF21: fibroblast growth factor 21
FXR: farnesoid X receptor
GalNAc: N‐acetylgalactosamine
GLP‐1: glucagon‐like peptide‐1
HCC: hepatocellular carcinoma
KRAS: Kirsten rat sarcoma viral oncogene homolog
LDL: low‐density lipoprotein
MAPK/ERK: mitogen‐activated protein kinase/extracellular signal‐regulated kinase
MASH: metabolic dysfunction‐associated steatohepatitis
NFE2L2: nuclear factor, erythroid 2–like 2 (NRF2)
PLS: prognostic liver signature
PRDX2: peroxiredoxin 2
ROS: reactive oxygen species
STAT3: signal transducer and activator of transcription 3
TERT: telomerase reverse transcriptase
TP53: tumor protein p53
VEGF: vascular endothelial growth factor

## 1 Background

Hepatocellular carcinoma (HCC) remains a significant global health concern, as a leading cause of cancer‐related death worldwide. Although the incidence of virus‐associated HCC is decreasing due to effective antiviral treatments, metabolic dysfunction‐associated steatohepatitis (MASH) has become a major cause of liver cancer, particularly in high‐income regions [1, 2, 3]. Sedentary lifestyles, type 2 diabetes, and obesity create a metabolic environment that promotes inflammation, chronic liver damage, and ultimately, malignant transformation. The urgent need for strategies targeting early carcinogenic processes is underscored by the fact that, despite advances in systemic therapies such as combinations of VEGF inhibitors and immune checkpoint blockade, therapeutic options largely fail to prevent HCC development or recurrence [4].

Oxidative stress and dysregulated redox signaling are key mechanisms linking metabolic liver disease to HCC. In addition to causing DNA damage, long‐term accumulation of reactive oxygen species (ROS) activates proliferative and survival pathways that drive carcinogenesis [5]. In this context, thiol‐dependent antioxidant enzymes known as peroxiredoxins have emerged as context‐dependent regulators of tumor growth, cell survival, and proliferation. Specifically, PRDX2 has been identified as a tumor‐promoting factor in ovarian, lung, breast, and colorectal cancers [6, 7, 8, 9]. Studies have shown both protective and tumor‐promoting associations, but its role in liver disease remains debatable [10, 11].

**Fig. 1:**
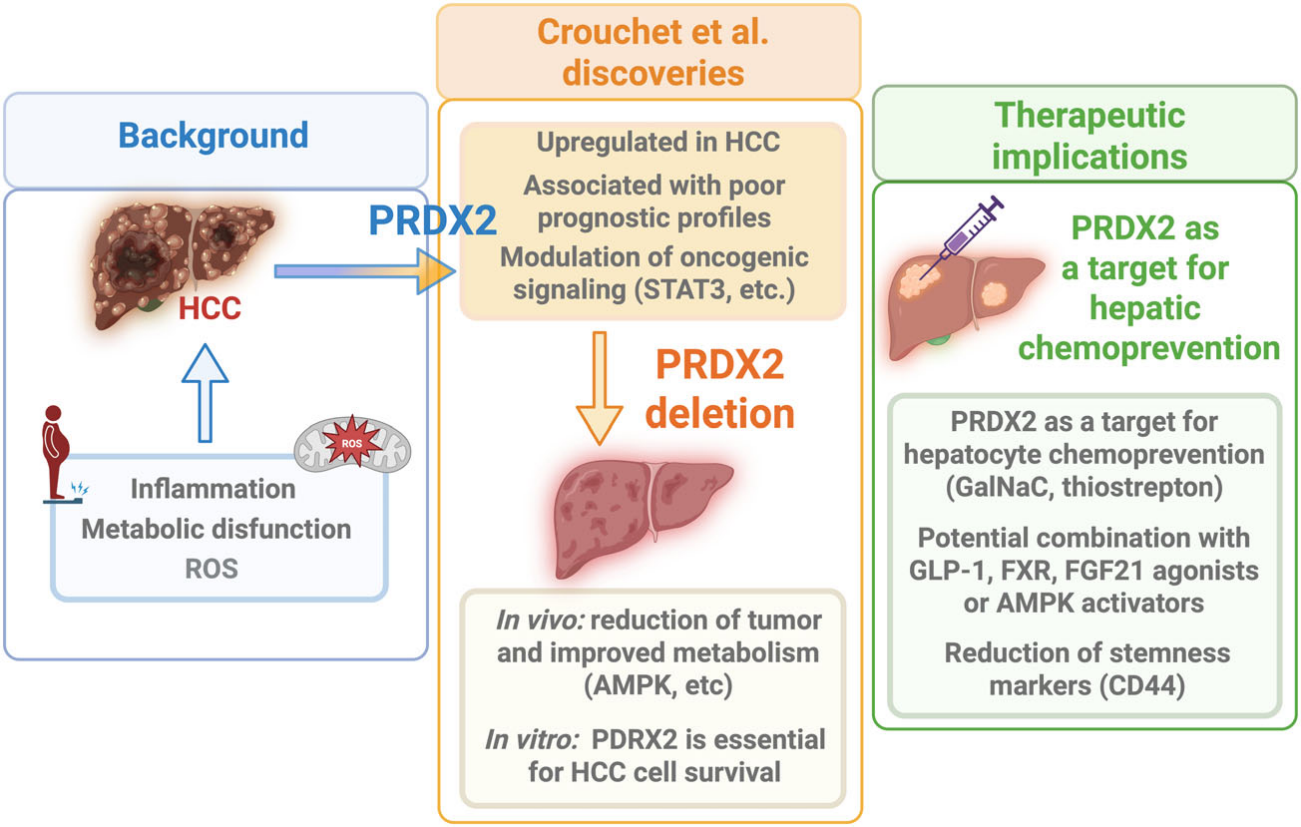
Role of PRDX2 in HCC and therapeutic implications. (Left) Background of HCC etiogenesis. Chronic inflammation, metabolic dysfunction, and elevated reactive oxygen species (ROS) promote HCC development. (Middle) Crouchet et al. show that PRDX2 is upregulated in HCC and correlates with poor prognostic profiles, partly through modulation of oncogenic signaling pathways, such as STAT3 and MAPK/ERK. Genetic deletion of PRDX2 leads to reduced tumor burden and improved metabolic regulation *in vivo*, and demonstrates that PRDX2 is essential for HCC cell survival *in vitro*. (Right) Therapeutic implications. Crouchet et al.'s findings corroborate the role of PRDX2 as a potential target for hepatic chemoprevention (e.g., GalNAc conjugation, thiostrepton) and combination with GLP‐1, FXR, FGF21 agonists, or AMPK activators.

Crouchet et al. offer a thorough mechanistic explanation for this discrepancy using an integrated, multidisciplinary experimental approach: they combine several *in vivo* models of MASH‐driven HCC, a cell‐based model of the clinical prognostic liver signature (PLS), and genome‐wide transcriptomics from human liver samples. In these study PRDX2 emerges as a key node linking oncogenic signaling and impaired metabolic homeostasis. Its expression is consistently higher in HCC than in the surrounding nontumorous liver tissue, correlates with poor‐prognosis PLS profiles, and is enriched in epithelial and progenitor‐like liver cell populations (Fig. 1, left panel).

## 2 Mechanistic insights

Crouchet et al. used multiple complementary experimental models to investigate the role of PRDX2 in metabolic dysfunction‐driven hepatocarcinogenesis (Fig. 1, middle panel). In the DEN/CDA‐HFD mouse model, hepatocyte‐specific Prdx2 deletion significantly reduced tumor burden, improved liver function, and partially ameliorated steatosis, while fibrosis remained largely unchanged. These mice showed restored AMPKα activation and increased expression of lipid and bile acid transporters, including *Scarb1*, *Abca1*, and *Abcg5/8*, along with reduced LDL cholesterol, indicating improved metabolic homeostasis. Transcriptomic profiling revealed suppression of key proliferative and oncogenic pathways (STAT3, MAPK/ERK, KRAS, and cell‐cycle signaling) together with decreased stemness markers and a shift in the PLS from poor‐to better‐prognosis profiles.

Moreover, *in vitro* models further clarified the cell‐type‐specific roles of PRDX2. In human hepatocytes, PRDX2 inhibition was well tolerated and restored AMPK activation, reduced lipid accumulation, and suppressed STAT3 and ERK signaling, supporting the idea that nonmalignant hepatocytes can tolerate redox modulation. In contrast, HCC cell lines showed a strict dependence on PRDX2's peroxidase activity for survival and malignant phenotypes. Clonogenicity and tumor‐sphere formation were restored only by wild‐type PRDX2, and not by a catalytically inactive C51S mutant, highlighting the essential role of PRDX2's antioxidant function in tumor initiation. This tumor‐selective dependency aligns with complementary evidence showing that PRDX2 stabilizes β‐catenin by preventing its oxidative inactivation, thereby sustaining Wnt/β‐catenin transcriptional activity and promoting stemness and tumor‐propagating capacity [10].

These findings were validated in cell line‐derived xenografts, where PRDX2 knockout impaired tumor growth and reduced AKT and ERK activation, and in patient‐derived HCC spheroids, which confirmed selective apoptosis of malignant cells while sparing nonmalignant hepatocytes. Across models, RNA‐Seq and PLS analyses consistently showed that PRDX2 modulates metabolic, proliferative, oncogenic, and stemness pathways, linking oxidative stress management directly to hepatocarcinogenesis.

Together, these models provide a mechanistic framework demonstrating that PRDX2 acts as a key metabolic and redox regulator. It amplifies oncogenic signaling in hepatocytes under metabolic stress, supports malignant transformation, and represents a promising target for liver‐directed chemoprevention. In this framework it is important to mention that, conversely, PRDX2 deficiency in metabolic MASH models could aggravate steatohepatitis and liver injury in female mice under metabolic stress, demonstrating that PRDX2 can exert hepatoprotective functions depending on sex and disease stage [11].

## 3 Therapeutic perspectives and translational implications

With the rising incidence of MASH‐related HCC, interventions targeting early carcinogenic pathways are becoming more relevant. The study by Crouchet et al. identifies PRDX2 as a promising target for chemoprevention (Fig. 1, right panel). Using GalNAc‐conjugated siRNAs, the authors demonstrate hepatocyte‐specific PRDX2 knockdown even after metabolic disease is established, resulting in reduced tumor initiation.

Targeting PRDX2 could be further enhanced by combining it with therapies that restore hepatocyte metabolic homeostasis. Agents such as GLP‐1 receptor agonists, FXR agonists, FGF21 analogs, or direct AMPK activators may act synergistically with PRDX2 inhibition by lowering upstream oxidative stress. In addition, PRDX2 inhibition appears to suppress the expression of stemness marker CD44, potentially reducing recurrence risk, a critical clinical issue given that up to 70% of patients relapse within 5 years after curative resection [2].

Pharmacological targeting of PRDX2 is also promising. The translational potential of redox‐directed therapy is demonstrated by thiostrepton, which selectively induces apoptosis in cancer cells while sparing nonmalignant hepatocytes, as demonstrated in patient‐derived HCC spheroids.

A particularly interesting aspect is the dual context of PRDX2 targeting in relation to AMPK. In hepatocytes, AMPK‐mediated metabolic homeostasis is supported by PRDX2, helping to restore lipid metabolism and reduce steatosis. In tumor cells, PRDX2 inhibition triggers mitochondrial and ER stress, decreasing proliferation and promoting apoptosis independently of AMPK. This dual mechanism allows PRDX2 targeting to simultaneously impair tumor survival while preserving normal liver metabolism, as observed in mouse MASH/HCC models and patient‐derived spheroid. Looking ahead, it will be important to characterize how PRDX2 interacts with key HCC drivers, such as TERT, TP53, CTNNB1, and NFE2L2, and to define the optimal therapeutic window for intervention. This is particularly relevant for patients with advanced metabolic dysfunction, where early targeting of PRDX2 could provide effective chemopreventive benefits before the onset of irreversible liver damage.

However, preclinical data indicate that PRDX2 deficiency may worsen MASH in female mice, highlighting sex‐specific risks [11]. Thus, future therapeutic strategies should carefully define timing, patient sex, and metabolic context to balance chemopreventive efficacy against hepatotoxicity.

## 4 Conclusions

What Crouchert et al. contribute as novel insights to the existing knowledge of HCC and PDRX2, is summarized in Fig. 1. Taken together, these findings indicate that PRDX2 is a promising biomarker and a feasible hepatocyte‐directed target for chemoprevention in metabolic liver disease. By clarifying how oxidative stress intersects with metabolic dysfunction, Crouchet et al. demonstrate that redox modulation targeting PRDX2 has the potential to reduce HCC risk and improve patient outcomes. These findings, together with other previous evidence, highlight the importance of understanding the interplay between metabolism and HCC development, showing that PRDX2 can be both hepatoprotective and tumor‐promoting depending on the metabolic and sex‐specific context. This demonstrates that PRDX2‐targeted strategies could be highly promising, but must be applied with precise molecular stratification to maximize therapeutic benefit while minimizing risk.

## 6 Conflict of interest

The authors declare no conflict of interest.

## 7 Author contributions

NG‐U, MLM‐C, and CC wrote and edited the commentary.
